# Fronto-Subcortical Circuits for Cognition and Motivation: Dissociated Recovery in a Case of Loss of Psychic Self-Activation

**DOI:** 10.3389/fpsyg.2018.02781

**Published:** 2019-01-23

**Authors:** Rodrigo Riveros, Serge Bakchine, Bernard Pillon, Fabrice Poupon, Marcelo Miranda, Andrea Slachevsky

**Affiliations:** ^1^Department of Psychology, University of Southern California, Los Angeles, CA, United States; ^2^Brain and Creativity Institute, University of Southern California, Los Angeles, CA, United States; ^3^Service de Neurologie, Centre Hospitalier Universitaire de Reims, Reims, France; ^4^Federation de Neurologie, Hôpitaux Universitaires Pitié Salpêtrière, Paris, France; ^5^Service de Neuroradiologie du Pr Marsault, Hôpital Universitaire Pitié Salpêtrière, Paris, France; ^6^Neurodegenerative and Movement Disorders Unit, Department of Neurology, Clinica Las Condes, Santiago, Chile; ^7^Geroscience Center for Brain Health and Metabolism, Santiago, Chile; ^8^Memory and Neuropsychiatric Clinic, Department of Neurology, Hospital del Salvador, Universidad de Chile, Santiago, Chile; ^9^Neuropsychology and Clinical Neuroscience Laboratory (LANNEC), Physiopathology Department, ICBM, Neurosciences Department, East Neuroscience Department, Faculty of Medicine, Universidad de Chile, Santiago, Chile; ^10^Center for Advanced Research in Education, Universidad de Chile, Santiago, Chile; ^11^Servicio de Neurología, Deprtamento de Medicina, Clínica Alemana, Universidad del Desarrollo, Santiago, Chile

**Keywords:** motivation, apathy, self-activation, dysexecutive, prefrontal, basal ganglia, cortico-subcortical circuits

## Abstract

In humans and non-humans primates, extensive evidence supports the existence of subcortico-cortical circuits for cognition and behavior. Lesions studies are critical to understand the clinical significance of these functionally segregated circuits. Mapping these circuits from lesion studies is difficult given the heterogeneous etiology of the lesions, the lack of long-term and systematic testing of cognitive and behavioral disturbances, as well as the scarcity of neuroimaging data for identifying the precise location and extent of subcortical lesions. Here, we report the long-term follow-up study of a patient who developed a loss of psychic self-activation associated to a dysexecutive syndrome following resuscitation from cardiac arrest. Neuroimaging revealed extensive bilateral lesions in the putamen, with a relative spare of the caudate, and exhibiting a dorsoventral gradient that was predominantly rostrally to the anterior commissure and spared most of the ventral striatum. In comprehensive neuropsychological and neuropsychiatric assessments, we observed dissociation between the improvement of the self-activation deficits and the stability of the dysexecutive syndrome. The pattern of recovery after this lesion lends support to current models proposing the existence of two main subcortico-cortical circuits: a dorsal circuit, mostly mediating cognitive processes, and a ventral circuit, implicated in motivation.

## Introduction

Loss of psychic self-activation (LPSA) is a syndrome characterized by striking reduction in spontaneous motion and speech, almost complete lack of initiative, absence of spontaneous mental activation of any kind, subjective “mental emptiness,” loss of interest for previously motivating activities, and apparent emotional flatness or poor expressiveness of affect (Laplane et al., 1984; De Witte et al., 2008). Importantly, the lack of spontaneous activation is temporarily reversible by external stimulation (Poncet and Habib, 1994). This syndrome has been referred to by various names, including auto-activation deficit (Laplane and Dubois, 2001), athymhormia (Habib, 2004), psychic akinesia, and reversible inertia (Laplane et al., 1984). LPSA has been often associated with dysexecutive syndrome (Laplane, 1990), although some patients show isolated behavioral disorder without cognitive dysfunction (Mori et al., 1996). Psychiatric symptoms, such as obsessive-compulsive-like behaviors, are also a feature of LPSA syndrome (Laplane et al., 1989). Long-term follow-up studies of LPSA have shown that the radical impairment in auto-initiated action do not significantly recover during the follow-up period (Poncet and Habib, 1994; Kaphan et al., 2014), and furthermore, some patients demonstrate a clear deterioration (Habib, 2004).

LPSA has been described to include bilateral lesions of the basal ganglia (BG), frequently affecting the caudate, pallidus and putamen (Bhatia and Marsden, 1994; Laplane and Dubois, 2001), and has been explained by a disruption of a frontal-subcortical circuit that contributes to motivation (Levy and Dubois, 2006; Schmidt et al., 2008). However, which structures are critically involved in LPSA remains unclear (Benke et al., 2003). The difficulty in mapping the brain structures related to LPSA is partly due to that previous studies have involved heterogeneous groups of LPSA patients whose symptoms were caused by different lesion etiologies with considerable anatomical variability. Moreover, isolated cases often lacked of systematic evaluation of the neuropsychological disturbances and topographical distribution of the lesions. Consequently, the specific neurobehavioral roles of anatomically defined regions of the BG are not as well established as the mechanism of cognitive impairment following BG lesions (Benke et al., 2003). Given this, there is a need for more case studies, with comprehensive neuropsychological and neuropsychiatric assessments, as well as neuroimaging data, in order to advance the understanding of LPSA and, more broadly, the motivational and cognitive deficits following injuries in fronto-subcortical structures (Moretti and Signori, 2016).

Here, we report the case of a patient suffering of LPSA associated to a dysexecutive syndrome following resuscitation from cardiac arrest. Written informed consent was obtained from the patient for the publication of this case, and for the identifiable information. The 3-year follow-up study, comprising neuropsychological and neuropsychiatric assessments, revealed a significant regression of the behavioral symptoms without concomitant recovery of the cognitive syndrome. Concurrent neuroimaging studies, comprising computerized tomography (CT), magnetic resonance imaging (MRI), and single-photon emission computed tomography (SPECT), showed bilateral damage to the BG, predominately located in the antero-dorsal part of the striatum with relative sparing of the ventral striatum, as well as prefrontal hypoperfusion. The pattern of recovery and the location of the lesion lend support to the model of functionally segregated fronto-subcortical circuits involved in cognition and motivation, validating similar models described in humans and non-humans primates (Haber, 2003; Choi et al., 2012).

## Case Report

LD is a right-handed, 26-year-old male, with 8 years of formal education, working as a kitchen aide. He had no medical history, but had been addicted to heroin for approximately 1 year. He suffered a sudden circulatory collapse of unknown duration due to a heroin overdose. He remained in a post-anoxic coma for 15 days. Upon awakening from the coma, LD’s behavior was characterized by significant inertia, lack of drive and complete loss of self-activation. In the absence of stimulation, LD neither talked, nor initiated any activity. He did not spontaneously complain about his state, although he acknowledged being ill and having voice, language, and memory problems. After direct questioning, he declared that he felt complete mental emptiness.

In the neurological examination LD showed a major impairment in his speech. His voice was aprosodic, hypophonic and characterized by accelerated articulation that led to an almost unintelligible speech. However, he was able to temporarily raise the volume of his voice upon request. The examination also revealed bilateral hyperreflexia. A psychiatric evaluation indicated low anxiety level, and marked flattened affect. When asked about his feelings and emotional reactions, LD declared to have none, but that he might have some if he were to encounter exceptionally intense situations. However, it was not possible to observe or provoke any sign of such reactions. LD reported non-significant depressive symptoms, and presented mild compulsive counting and checking.

One year following symptoms onset, forelimb dystonic syndrome appeared predominately in the left arm. The neurological examination showed preserved motor strength, brisk reflexes with a bilateral Babinski sign, dystonia and an intense akinetic syndrome without rigidity. Dystonia was particularly severe in the left hand, which was kept in a fist posture, and in the left foot, which exhibited hyperextended toes. He showed a pushover reaction toward his back when in a standing position. LD was very slow to initiate gait, which was slowed down and disturbed by akinesia, dystonia and freezing. His face was hypomimic with loss of spontaneous blinking and facial dystonia (so-called, sardonic smiling). Ocular movements were characterized by slow saccades.

Various treatments were attempted during the first 15 months following symptoms onset. LD was treated with an increasing dosage of carpipramine (50 – 150 mg/day), which was discontinued after 4 months due to the absence of any change in the patient’s behavior. Clonazepan (2 mg/day) led to an amelioration of dystonia. Levodopa was prescribed to treat the LPSA syndrome, and the dosage was progressively increased to a maximum of 750 mg/day, however, it was discontinued since no clinical change was observed. Fluvoxamine treatment was also used (100 mg/day) for 4 months, without noticeable change.

Three years following symptoms onset, LPSA had partially regressed. LD could carry out spontaneous motor activities, such as choosing movies to watch and going shopping regularly. He reported increased emotional response and mental activity. Notably, he also reported having dreams again. He described no longer performing compulsive behaviors, or having obsessive thoughts. Although he was able to spontaneously carry out many simple everyday living activities, he still had significant difficulties in planning and performing more complex actions, for which he depended on external control. His speech was less hypophonic, less accelerated, and more intelligible compared to previous neurological examinations. He still presented facial dystonia. Despite the persistence of asymmetric dystonic syndrome that was more accentuated in the left hand and foot, his motor performance strikingly improved, with quicker and more coordinated movements.

### Neuroimaging Study

A CT scan was performed within the first 15 days after the cardiac arrest. Fourth months after the cardiac arrest, LD received a brain MRI and a SPECT. A second MRI with sequences for 3-D reconstruction in order to precise the localization of the striatal damage was performed 3 years following symptoms onset.

**FIGURE 1:**
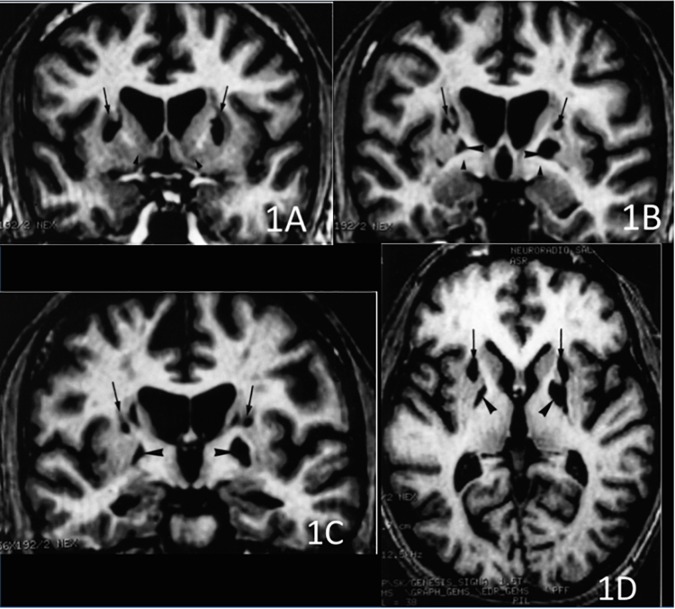
3D T1-weighted axial and coronal sections at 3 different rostro-caudal levels of the lesion of the basal ganglia. **(A)** Coronal section passing at the level of the head of the caudate nucleus and the ventral striatum. The lesions involved the dorsal striatum (arrows) while the ventral striatum was apparently spared (arrowheads). **(B)** Coronal section passing at the level of the anterior commissure. Infarction was located in the upper part of the putamen (arrows) and the globus pallidus (large arrowheads). The head of the caudate nucleus was atrophic. The ventral pallidum was spared (small arrowheads). **(C)** Coronal section passing at the level of the mammillary bodies. The lesion involved the upper part of both putamen (arrows) and the globus pallidus (arrowheads), bilaterally. **(D)** Axial section showing the rostro-caudal gradient of striatal lesion, which predominated in the rostral “associative” part of the striatum (arrows), and the lesion of the globus pallidus (arrowheads).

**FIGURE 2:**
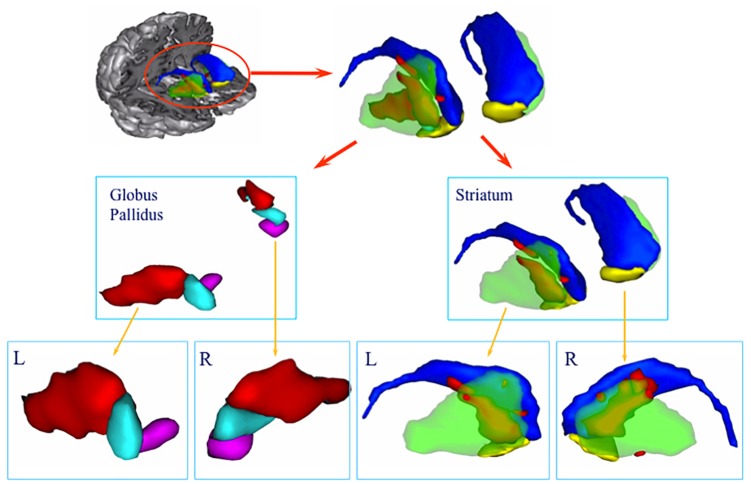
3D volume rendering of basal ganglia lesion. The lesion is represented in red. Green: putamen, blue: caudate nucleus, yellow: ventral striatum, light blue: dorsal pallidum, purple: ventral pallidum. Lower row: lateral view of the left **(L)** and right **(R)** globus pallidus and striatum. Note the predominance of the lesion in the anterodorsal part of the striatum.

The CT scan showed diffuse cerebral swelling. The first MRI showed extensive bilateral lesions affecting the lenticular nucleus and other partial lesions in the caudate nucleus, as well as in adjacent white matter between these structures. The SPECT scan revealed bilateral prefrontal hypoperfusion. The second MRI allowed a more precise localization of subcortical lesions revealing that the lesions were larger in the putamen than in the caudate and had a clear dorsoventral gradient that was predominantly rostrally to the anterior commissure and spared most of the ventral striatum. The same pattern was found in the pallidus, in which the lesions affected more the dorsal than the ventral region. Additionally, the MRI scan showed mild lesions of the anterior and antero-superior periventricular white matter in the frontal region and moderate atrophy of both hippocampi and the left hemisphere of the cerebellum (see Figures 1, 2).

### Neuropsychological and Neuropsychiatric Assessments

LD completed a comprehensive neuropsychological battery 4, 15, and 36 months after the cardiac arrest, as well as neuropsychiatric assessments 15 and 36 months after the symptoms onset. More details about the assessments are presented in the Supplemental Information section.

In the first neuropsychological assessment, LD’s lack of initiative was a considerable limitation and it was only partially completed at that time. In the second and third neuropsychological assessments, the primary disorder was a prominent dysexecutive syndrome with reduced verbal fluency, cognitive slowing and considerable inertia. LD’s performance on the California Sorting Test provided a striking illustration of his deficits, performing similarly to patients with focal damage to the frontal lobes (Delis et al., 1992). Other major disturbances were a reduced global cognitive capacity, and impaired working and episodic memory. The three consecutive neuropsychological evaluations showed consistently below expected performance in global cognitive efficiency and episodic memory, as well as more severe impairments on executive functions, with a slight improvement in conceptual capacities and lexical evocation in the third evaluation (see Table 1).

**Table 1:** Results of neuropsychological evaluation performed at months 4, 15, and 36 from onset.

Neuropsychological domain	Time from onset (in months)		
	4	15	36
**Global cognitive efficiency**			
Mini-mental state examination (28 ± 2)	16	22	20
Mattis dementia rating scale (140 ± 4)	111	124	127
WAIS-R verbal scale (100 ± 15)	NT	79	84
Raven’s progressive matrices (100 ± 15)	NT	99	92
**Attention and working memory**			
Forward digit span WAIS-R (6 ± 1)	6	7	6
Backward digit span WAIS-R (5 ± 1)	3	3	4
**Verbal learning –** Free and cued selective reminding test			
Immediate cued recall (15 ± 0.5)	NT	13	15
Total free recall (39 ± 5)	NT	15	13
Sum of free and cued recall (46 ± 2)	NT	40	40
Free delayed recall (14 ± 1.5)	NT	5	3
Sum of free and cued delayed recall (15 ± 1)	NT	14	14
Recognition (15.9 ± 0.2)	NT	15	16
**Flexibility –** Trail making test			
Form A (32 ± 16)	68	50	61
Form B – Form A (37 ± 70)	243	122	107
**Inhibition of interferences –** Stroop test			
Reading of words (108 ± 20)	NT	60	56
Naming of colors (80 ± 15)	NT	44	38
Color-words interference (45 ± 10)	NT	16	29
**Verbal fluency**			
Category fluency (23 ± 5)	NT	8	15
Letter fluency (16 ± 5)	NT	3	9
**Conceptual elaboration and shifting –** Modified Wisconsin card sorting test			
Categories (5 ± 1)	NT	2	3
Perseverative errors (2 ± 1)	NT	4	3
**Conceptual elaboration and shifting –** California card sorting test			
Condition 1- attempted sorts (20 ± 2)	NT	14	15
Correct sorts (15 ± 2)	NT	8	12
Perseverations (1 ± 1)	NT	6	2
Condition 2- correct rule names (14 ± 2)	NT	2	3
Condition 3- abstract cues (20 ± 2)	NT	17	22
Explicit cues (23 ± 1)	NT	24	24


*Numbers in brackets indicate the expected mean ± SD in healthy adults, according to the authors of each test. NT, not tested at this time point. Mini-mental state examination (Folstein et al., 1975), Mattis dementia rating scale (Mattis, 1976); WAIS-R, Revised Wechsler adult intelligence scale (Wechsler, 1981); RPM, Raven’s progressive matrices (Raven, 1981); FCSRT, Free and cued selective reminding test (Grober et al., 1988); Trail making test (Reitan, 1958); verbal fluency tests, Stroop test (Stroop, 1935); MWCST, Modified Wisconsin card sorting test (Nelson, 1976); CST, California sorting test (Delis et al., 1992).*

**FIGURE 3:**
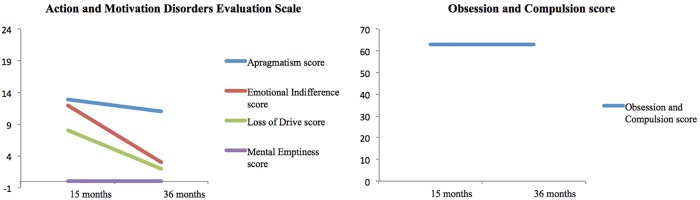
Results of neuropsychiatric evaluation conducted at 15 and 36 months after the onset. In the Action and Motivation Disorders Evaluation Scale (Habib, 1995), the maximum score for each measure is 24. In this scale, higher scores reflect higher impairment. In the Obsession and Compulsion Evaluation Scale (Frankel et al., 1986), subjects without obsessive or compulsion symptoms score below 40.

In contrast to the stability of the cognitive disorder, the neuropsychiatric assessment revealed a progressive recovery over time (see Figure 3). The emotional indifference and loss of drive scores improved by approximately 75% between the two examinations. The apragmatism score improved by 15%. The Obsession and Compulsion Evaluation Scale scores did not change between the two examinations, with both scores reflecting the presence obsessive compulsive-like symptoms.

## Discussion

This report presents a case of LPSA and dysexecutive syndrome following a cardiac arrest. 3 years after the cardiac arrest, LD exhibited dissociated improvement of his condition. His drive, motor initiative and emotional symptoms greatly improved, whereas the cognitive deficits remained, except for a slight amelioration of his lexical fluency and conceptual capabilities. Of importance, MRI scan revealed bilateral lesions predominantly in the putamen with a clear a dorsoventral gradient sparing most of the ventral striatum. The dorsovental gradient was also found in the pallidus. The association between the topography of LD’s lesion in the BG and the evolution of his cognitive and behavioral symptoms is consistent with models proposing the functional segregation of fronto-subcortical circuits involved in motivation and cognition.

Anatomical studies have shown the segregation of fronto-subocortical circuits. Heimer and Wilson (1975) and Heimer (2003) described the ventral striatal-pallidal system after demonstrating that the BG, including the olfactory tubercle, extends to the ventral surface of the mammalian brain. The ventral striatum includes the nucleus accumbens, the medial and ventral portions of the caudate nucleus and putamen, and the striatal cells of the olfactory tubercle (Heimer, 1978; Haber, 2003). The medial-dorsal nucleus of the thalamus, rather than the ventrolateral or the ventral anterior nuclei, is the primary nucleus of the ventral striatal-pallidal system, providing indication of segregated cortico-subcortical re-entrant circuits throughout the BG to the motor and prefrontal cortex (Heimer, 2003). Projections from the frontal cortex form a functional gradient of input from the ventromedial sector through the dorsal striatum, with the medial and orbito-frontal cortex in the ventromedial region, and the motor cortex terminating in the dorsolateral region (Haber, 2003). Based on those neuroanatomical findings, Alexander et al. (1986) suggested the existence of five cortico-subcortical circuits involved in cognition, behavior, and motor functions in primates and extrapolated their existence to humans. Three of these circuits have been implicated in high-order human behaviors: the prefrontal dorsolateral cortex circuit, the orbito-lateral frontal cortex circuit and the anterior cingulate cortex circuit (Cummings, 1993; Bonelli and Cummings, 2007).

The dissociation between cognition and behavior within these circuits have been supported by neuroimaging studies in healthy humans (Leh et al., 2007; Di Martino et al., 2008), and patients with neuropsychiatric diseases (MacDonald et al., 2011; Pauls et al., 2014). Draganski et al. (2008) in a tractography study showed that segreated and overlapping connections from cortical sites to the specific portions of the BG, underlying the parallel processing and the integration of motivational aspects of decision-making and cognitive contextual representations. On obsessive-compulsive disorder patients, a resting-state functional connectivity study found a dissociation within fronto-subcortical circuits for cognitive flexibility and goal-directed planning (Vaghi et al., 2017). Within the striatum, there is extensive evidence of dissociable functions, with the ventral portion involved in processing motivation salience, and the dorsal part more involved in cognitive control (Doherty et al., 2004; Jensen and Walter, 2014).

Lesion studies are less frequent; nonetheless, they offer an invaluable opportunity to understand the functional significance of these circuits in motivation and cognition. Dissociation between motivation and cognitive deficits has been reported in very few patients, besides LD. All patients previously reported had lesions that were restricted to a part of the striatal system, with an unequal distribution of damage along the ventral-dorsal axis. Mori et al. (1996) described a patient with bilateral necrosis of the ventral pallidus showing only a behavioral disorder and not associated dysexecutive syndrome. Haaxma et al. (1993) described a patient with bilateral lesions of the dorsal pallidus who presented with a dysexecutive syndrome without loss of self-activation. Miller et al. (2006) described a patient with a bilateral lesion in the ventral pallidus secondary to circulatory collapse due to an overdose of methadone. After that episode, the patient developed symptoms suggestive of severe depression, including depressed mood, anhedonia, low energy, feelings of hopelessness and guilt, poor self-esteem, social isolation, increased sleep, and a 20 lb.-weight gain over the ensuing year. Neuropsychological evaluation revealed intact cognitive functioning, including executive functions. In LD, the delayed regression of LPSA and the stability of the dysexecutive deficits could be explained by the presence of the dorsal-ventral gradient of damage to BG, with varied degrees of damage to the striatum and the pallidus secondary to anoxia. The damage to the ventral striatum and pallidus did not result in necrosis, explaining the clinical improvement that was observed over time (Kesavadas et al., 2009). Thus, the persistence of the dysexecutive syndrome could be due to a predominance of dorsal lesions, while a relative sparing of ventral regions could explain the improvement of motivation. This case, along with the few cases mentioned above, suggest the involvement of ventral circuits in motivation and the dorsal areas on cognition.

LD also presented a delayed and moderate postural dystonic syndrome that was predominant in the left extremities. A long delay between BG anoxia and the development of a movement disorder is not unusual (Bhatt et al., 1993). Dystonia can be explained by lesions in the sensory-motor striatum, including the right putamen posterior to the anterior commissure and the globus pallidus (Lehericy et al., 1996). The asymmetry of the lesions, predominantly in the right sensory-motor striatum, is consistent with the asymmetry of the dystonic syndrome. The motor symptoms observed in LD could be partially concealing the behavioral manifestations of the improvement on motivation, and therefore, the magnitude of the recovery should not be understated.

This single-case study has limitations. First, the patient had a diffuse injury as reflected in the bilateral prefrontal hypoperfusion and white matter involvement. This is an inherent limitation of single case studies with this level of specificity. Nevertheless, it does not prevent this study to contribute with evidence of validity to theoretical models of fronto-subcortical circuits. Indeed, prefrontal hypoperfusion has been also reported as part of the neural correlates of LPSA (Bogousslavsky et al., 1991; Engelborghs et al., 2000). In addition, these lesions and their locations did not seem to play a major role in LD’s symptoms because the lesions did not compromise the white matter pathways connecting the BG and the frontal cortex (Lehericy et al., 2004; Thiebaut de Schotten et al., 2011). Second, despite presenting evidence of the dysexecutive deficits and the regression of LPSA, we were unable to present longitudinal data using functional neuroimaging methods, such as Functional Dopamine-Transporter SPECT Imaging (DaT). This might have strengthened the correlation between brain functioning and the dissociation in the improvement of motivation over cognitive performance. The availability of functional neuroimaging methods, such as DaT will allow a more comprehensive evaluation of the physiopathology of LPSA *in vivo*, and a closer examination of this hypothesis. Third, the distinction between depression and LPSA has proven to be difficult to make, since anhedonia and lack of initiative are key symptoms of depression, as well as they are present in LPSA (Laplane and Dubois, 1998). Nevertheless, in our case, the patient did not report significant sadness or negative thoughts. Also, the lack of initiative in LPSA was reversible when the patient is instructed. These are two key differences between LPSA and depression, either after a cardiac arrest (Naber and Bullinger, 2018), or after a vascular accident (Habib, 2004). In addition, because of the lack of response to antidepressants, such as fluvoxamine, it seems unlikely that the motivational symptoms can be explained uniquely by depression. Instead, the mild depressive symptoms observed are a manifestation of the motivational disorder. Nevertheless, temporally distant effects of the pharmacological agents used during LD’s treatment cannot be ruled out.

This single-case study helps to illustrate more subtle clinical signs revealing the specialization of these circuits (Levy and Dubois, 2006), and it contributes to the current understanding of motivation-cognition interactions by illustrating how motivation, as a construct leading to value-oriented behavior, can be distinguished from, and integrated to affect, attention and higher cognitive processes (Braver et al., 2014).

## Concluding Remarks

The fronto-subcortical hypothesis of LPSA syndrome represents a promising explanatory model that awaits confirmation from more precise lesion studies (Benke et al., 2003). The present case, together with those from Haaxma et al. (1993) and Mori et al. (1996) lend support to the hypothesis of different parallel fronto-subcortical circuits in humans that are involved in cognition, behavior and motor functions, contributing to bridge the gap between experimental studies in animal and humans, and clinical research. Furthermore, these cases are consistent with models proposing a transfer of information along a ventral to dorsal gradient via circuits that span from emotional and motivational areas to decision making and executive control areas, and then to motor control areas (Haber et al., 2000; Haber, 2003). Only a few cases, such as LD, suggest the specialization of these circuits in humans, advancing our understanding of the functional significance of these circuits in purposeful behavior.

## Author Contributions

BP, SB, and AS designed the research. BP, FP, and AS performed the research. RR, BP, FP, MM, SB, and AS analyzed the data. RR and AS performed the literature review and wrote the manuscript.

## Conflict of Interest Statement

The authors declare that the research was conducted in the absence of any commercial or financial relationships that could be construed as a potential conflict of interest.
