# Supplementary material for: Fronto-Subcortical Circuits for Cognition and Motivation: Dissociated Recovery in a Case of Loss of Psychic Self-Activation
Source: Front Psychol. 2019 Jan 23;9:2781. doi: 10.3389/fpsyg.2018.02781 (PMC6352737; doi:10.3389/fpsyg.2018.02781)
Supplement: Supplementary file 1 [file Data_Sheet_1.docx]

Supplemental information.

Neuropsychological assessment.

Depending on the time of evaluation, the patient was assessed with some or all of the following tests or scales. Global cognitive performance was evaluated by Folstein's Mini-mental State Examination (MMSE) (Folstein, Folstein & McHugh, 1975), the Mattis Dementia Rating Scale (DRS) (Mattis, 1976), the Revised Wechsler Adult Intelligence Scale (WAIS-R) (Wechsler, 1981) and Raven's progressive matrices (RPM 38) (Raven, 1981). Attentional capabilities were evaluated using the attention subtest of the DRS and the Digit Span subtest of the WAIS-R. Verbal learning capabilities were assessed using the Free and Cued Selective Reminding Test (FCSRT) (Grober, Buschke, Crystal, Bang & Dresner, 1988). Executive functions were evaluated using the Trail Making test (Reitan, 1958), the Stroop test (Stroop, 1935), semantic and phonemic verbal fluency tests, and the Modified Wisconsin Card Sorting Test (MWCST) (Nelson, 1976). In addition to the MWCST, we used the California Sorting test (CST) (Delis, Squire, Bihrle & Massman, 1992), which allows for a more precise assessment of the level of impairment in conceptual abilities. In the CST, the patient sorts cards under three conditions: spontaneous, structured and cued sorting. The CST scores performance in several dimensions: initiation (evaluated by the number of attempted sorts, either correct or not), ability to identify and verbalize sorting rules from multiple stimulus properties and generation of accurate sorts (measured by the number of correct sorts) during spontaneous sorting. The test measures the ability to identify and verbalize sorting principles without having to transfer knowledge to action, the presence of verbal perseveration in the structured condition, the ability to comprehend abstract information to identify correct sorts and the capacity to use concrete feedback to regulate behavior in the cued sorting.

During the second evaluation (15 months following symptoms onset), the primary disorder was a prominent dysexecutive syndrome with reduced verbal fluency, cognitive slowing and considerable inertia (see Table 1). The patient’s performance on the CST provided a striking illustration of his deficits. In condition 1, the numbers of attempts was similar to that of controls (14 over 24, mean of controls: 16.2). However, the success rate was low (8 over 24, mean of controls: 14.2), and the number of perseverating answers was high. LD performed quite similarly to patients with focal damage to the frontal lobes (mean number of attempts 14.8; mean correct: 9.4), as described by Delis and collaborators (Delis et al., 1992). During follow-up evaluation (36 months following symptom onset), LD’s performances had improved slightly (15 attempts, 12 correct). However, LD failed markedly in condition 2, identifying only two rules out of 24. His score was considered highly pathological when compared with controls (mean: 14.9). Contrary to condition 1, LD’s score was much lower than scores obtained by frontal-damaged patients (mean: 7.8). LD performed better in condition 3, showing a normal ability to use explicit cues provided to him by the examiner but showing difficulties with abstract rules (abstract rules-LD: 17, controls: 22; explicit cues-LD: 24, controls: 23.7). These performances were similar to those that have been observed in frontal-damaged patients (17.4 and 21.4, respectively). LD improved his performance with abstract cues (22 out of 24 at the last evaluation). The other major disturbances were a reduced global cognitive capacity and impaired working (abnormal inverse digit span) and episodic memory (performance on FCSRT) (see Table 1). Of importance, the three consecutive neuropsychological evaluations showed below expected and stable performances in global cognitive efficiency, memory capacities and most executive functions, with a slight improvement in the conceptual capacities and lexical evocation during the last evaluation.

Neuropsychiatric assessment.

LPSA symptoms were assessed in the neuropsychiatric evaluation using The Action and Motivation Disorders Evaluation Scale (AMDES) (Habib, 1995), and the Obsession-Compulsion Evaluation Questionnaire (OCQ) (Frankel et al., 1986). The Action and Motivation Disorders Evaluation Scale (AMDES) is a three-part structured questionnaire. A family member completes the first part, the patient completes the second part, and the examiner completes the third part. Sub-scores are determined for apragmatism, emotional indifference, loss of drive and mental emptiness. The Obsession-Compulsion Evaluation Questionnaire (OCQ) with 40 questions completed by the patient with the help of the clinician (Frankel et al., 1986). The results of all these tests are shown in figure 3.
